# A whole-transcriptome analysis of differentially expressed genes, transcripts, and transcript usage in blood samples from Parkinson’s disease patients

**DOI:** 10.3389/ebm.2026.11099

**Published:** 2026-07-08

**Authors:** Sulev Koks, Mari Muldmaa, Jack Price, Luke Whiley, Maili Jakobson, Lewis Singleton, Denise Howting, Abigail L. Pfaff, Abha Chopra, Mark Watson, Katrin Sikk, Pille Taba

**Affiliations:** 1 Perron Institute for Neurological and Translational Science, Perth, WA, Australia; 2 Personalised Medicine Centre, Health Futures Institute, Murdoch University, Perth, WA, Australia; 3 Institute of Clinical Medicine, University of Tartu, Tartu, Estonia; 4 Department of Neurology, North Estonia Medical Centre, Tallinn, Estonia; 5 Curtin Medical School, Faculty of Health Sciences, Curtin University, Perth, WA, Australia; 6 Curtin Medical Research Institute, Curtin University, Perth, WA, Australia; 7 Dementia Centre of Excellence, enAble Institute, Curtin University, Perth, WA, Australia; 8 Centre for Computational and Systems Medicine, Health Futures Institute, Murdoch University, Perth, WA, Australia; 9 Institute of Biomedicine and Translational Medicine, University of Tartu, Tartu, Estonia; 10 Clinic of Neurology, Tartu University Hospital, Tartu, Estonia

**Keywords:** alternative splicing, biomarkers, gene expression profiling, Parkinson’s disease, RNA-seq

## Abstract

Parkinson’s disease (PD) is a complex neurodegenerative disorder with diverse molecular signatures that extend beyond the central nervous system. Peripheral blood serves as a minimally invasive source of transcriptomic biomarkers reflecting systemic inflammation, mitochondrial dysfunction, lysosomal impairment, and disrupted RNA processing—key pathways involved in PD. Long-read RNA sequencing with Oxford Nanopore Technologies (ONT) offers unprecedented detail of full-length transcripts, alternative isoforms, and RNA modifications, enabling more accurate detection of disease-related transcriptional changes. We conducted high-throughput ONT long-read RNA sequencing on blood samples from 145 individuals, including PD patients and age-matched healthy controls. RNA libraries were prepared using a cDNA-based protocol optimised for high-accuracy PromethION sequencing. Transcriptomes were quantified with ONT-specific pipelines, and analyses of differential gene expression (DGE), differential transcript expression (DEX), differential transcript usage (DTU), and alternative splicing were performed using ONT-aware tools such as DRIMSeq, DEXSeq, and stageR. Pathway enrichment linked disease-related changes to mitochondrial, lysosomal, and vesicle-trafficking pathways. Our analysis identified highly significant PD-associated transcriptional signatures in peripheral blood (SNCA, VPS13C, LRRK2, GRN, STAU1, NPTN, PARK7). Long-read sequencing uncovered extensive isoform-level dysregulation, including novel transcript variants (e.g., BCL2L2-PABPN1, SQSTM1) in pathways relevant to PD, such as autophagy and endolysosomal trafficking. DTU analyses revealed shifts in isoforms of LRRK2 and GBA1, indicating widespread disturbances in RNA processing. Enrichment analysis showed activation of molecular pathways linked to neurodegeneration. This study provides the largest long-read blood transcriptomic dataset in PD to date, demonstrating that ONT sequencing can resolve isoform-level changes and reveal systemic molecular signatures of PD. Our findings support the development of blood-based RNA biomarkers and the establishment of long-read transcriptomics as a transformative approach for genomic pathology in PD.

## Impact statement

The submitted work is important because it describes Oxford Nanopore sequenced long-read transcriptomic changes in Parkinson’s disease (PD) blood. The work advances the field by identifying novel transcripts related to PD. Our research highlights several new transcripts and splicing changes in transcript isoforms that could not be detected with short reads. This study demonstrates that long-read sequencing can uncover significant differences that other technologies cannot.

## Introduction

Parkinson’s disease (PD) is a progressive neurodegenerative disorder characterised clinically by bradykinesia, rigidity, tremor, and postural instability [1]. Pathologically, it involves the loss of dopaminergic neurons in the substantia nigra pars compacta (SNpc) and the buildup of α-synuclein–rich Lewy bodies [1, 2]. Over the past 2 decades, advances in human genetics - supported by genome-wide association studies (GWAS), research on monogenic families, and high-resolution sequencing - have revealed that PD is a complex condition with multiple distinct yet interconnected biological pathways [3, 4]. While fewer than 10% of patients have pathogenic monogenic variants, research on genes such as SNCA, LRRK2, VPS13C, PARK2, PINK1, PARK7, GBA1, and other risk loci has identified key mechanisms, including α-synuclein proteostasis, mitochondrial quality control, lysosomal and autophagic dysfunction, vesicular trafficking, and immune and inflammatory responses [3–5].

At the same time, the functional genomic understanding of these genetic networks has been somewhat delayed, and gene expression analysis in PD patients has not been very intensive. The complex interactions involved suggest that the underlying genomic mechanisms are likely multifactorial and intricate [6–10]. Multiple studies have used transcriptomic analyses to identify PD signatures, revealing distinct transcriptional patterns or splicing events in genes expressed in the basal ganglia [11, 12]. Due to the challenges of obtaining brain tissue from patients, there is a growing need for peripheral biomarkers. Analyses of transcriptomes from accessible tissues such as skin, keratinocytes, and blood, through the lens of CNS expression data from PD patients, have revealed notable differences between PD and control groups [7, 8, 13]. These findings underscore the importance of studying peripheral tissues not only as a means to find biomarkers for disease prediction but also to deepen our understanding of neurodegeneration.

Peripheral tissues offer a rich source of biomarkers for chronic neurological conditions, and most commonly, blood can be used for this purpose. Indeed, blood has been shown to provide a great source for RNA and transcriptomic profiling for clinical use, with a very good overlap with protein analysis, even in neurological conditions [13–15]. Serum amyloid A is a good example of a potential biomarker that shows changes in RNA and protein levels in PD patients. Many previous studies have performed whole-transcriptome analysis of blood from PD patients [16–22]. Interestingly, SNCA expression in the blood of PD patients is lower than in healthy controls [16]. In addition, the changes in the PD genes (e.g., GBA1, LRRK2) have been inconclusive. All these studies have been based on Illumina short reads, which have limitations in analysing transcriptome complexity. While the short reads are excellent to capture aggregated gene-based transcription signals, they are challenging to call individual transcripts of specific genes. It is possible to use short reads for more specific analytical workflows and to measure exonic or intronic reads, as well as long non-coding RNAs [21–23].

Gene expression analysis can target a single gene, a specific gene group, or all genes in the genome, serving as a valuable tool for biomarker or diagnostic development. This information is also essential for understanding the underlying pathological processes. Whole transcriptome profiling, also known as genome-wide gene expression analysis, provides the most comprehensive view of a biological system or its response to stimuli, making it invaluable for complex analyses. It can be performed in different forms. This method can be applied to various biosamples and environmental samples through meta-transcriptome analysis. It is crucial to understand the changes in RNA expression that arise directly from a disease-causing mutation or from the interaction of the mutated and dysfunctional gene product. Additionally, RNA profiling is particularly useful for characterising molecular changes associated with disease states, aiding in the identification of potential RNA-based biomarkers.

In the present study, we employed Oxford Nanopore long-read sequencing of blood RNA from patients with PD and healthy controls (HC). We used a whole-transcriptome protocol that captures both poly-A and non-poly-A RNA, providing a comprehensive profile of variable RNA isoforms. This method allowed us to accurately characterise differential expression at the levels of genes, transcripts, and transcript usage by gene. We also compared our findings with existing PD transcriptome studies, like the Parkinson’s Progression Markers Initiative (PPMI), to gain a broader understanding of the blood transcriptome in PD patients.

## Materials and methods

### Ethics, recruitment, sample collection and storage

PD patients were enrolled in this study from neurology outpatient clinics at four major hospitals in Estonia—North Estonia Medical Centre, Tartu University Hospital, East Tallinn Central Hospital, and Pärnu Hospital. All patients had previously been diagnosed according to the Queen Square Brain Bank (QSBB) criteria for Parkinson’s disease. For this study, we used samples from 145 subjects: 61 PD patients and 84 HCs.

Demographic data and medical history were collected (Table 1). Patients underwent comprehensive neurological and psychological evaluation using the following instruments: the Movement Disorders Society’s Unified Parkinson’s Disease Rating Scale (MDS-UPDRS) [24], Hoehn and Yahr Scale (HY) [25], Movement Disorders Society’s Unified Dyskinesia Rating Scale (UDysRS) [26], and Mini-Mental State Examination (MMSE) [27].

**TABLE 1:** General characteristics of the study cohort.

Group	Parkinson’s disease (PD)	Healthy controls (HC)
Total, n^1^	61	84
Male, n	25 (41.0%)	36 (42.9%)
Female, n	36 (59.0%)	48 (57.1%)
Total mean age, y (sd)	69.8 (9.6)	70.1 (7.6)
Male mean age, y (sd)	69.5 (8.1)	68.4 (11.3)
Female mean age, y (sd)	71.5 (7.3)	70.9 (8.0)
PD duration, y (sd)	7.9 (5.8)	-
Male duration, y (sd)	8.4 (6.6)	-
Female duration, y (sd)	7.7 (5.4)	-
AASO (sd)	61.4 (8.5)	​
YFSO (sd)	9.9 (8.7)	​
HYS (sd)	2.4 (0.6)	​
MMSE (sd)	27.4 (3.0)	​
L-DOPA, mg (sd)	534.4 (288.7)	​
LEDD, mg (sd)	784.9 (432.8)	​
Total UPDRS (sd)	47.63 (21.6)	​
Part III UPDRS (sd)	23.9 (13.3)	​
Part IV UPDRS (sd)	2.3 (3.5)	​
UDysRS (sd)	5.2 (7.8)	​

1 n, number of subjects; y, years; sd, standard deviation; AASO, age at symptom onset; YFSO, years for symptom onset; HYS, Hoehn and Yahr scale; MMSE, Mini-Mental State Examination; LEDD, Levodopa Equivalent Daily Dosage; UPDRS, Unified Parkinson's Disease Rating Scale; UDysRS, Unified Dyskinesia Rating Scale.

Levodopa equivalent doses were calculated as proposed by Tomlinson et al [28]. Age- and sex-matched control participants were recruited from the laboratory of the North Estonia Medical Centre. The inclusion criteria required that participants be outpatients without any known central nervous system disorders who were referred for routine blood tests. Individuals from the neurology, neurosurgery, hematology, oncology, and rheumatology clinics were excluded to avoid potential confounding effects related to their diagnoses or treatments.

Venous blood from all participants was collected into Tempus™ RNA tubes, and total RNA was isolated using the Tempus™ Spin RNA Isolation Kit (Applied Biosystems) according to the manufacturer’s standard protocol. Samples were then stored at −80 °C before being shipped on dry ice to the Medical Genomics Laboratory at Murdoch University (WA, Australia) for analysis.

The study received approval from the Human Research Ethics Committee of the Estonian Institute for Health Development, and written informed consent was obtained from all participants.

### Whole transcriptome sequencing using ONT

The bulk RNA-seq protocol employed an adapted SMART-seq assay that uses Oligo(dT) and Random Nonamers to target all transcripts and achieve whole-transcriptome coverage [29].

Before starting sample preparation, the RNA integrity was verified by checking the RIN score with the Agilent Tapestation. Ribosomal depletion was carried out using the NEBNext® rRNA Depletion Kit V2 (New England Biolabs), and the rRNA-depleted RNA was employed for cDNA synthesis with biotin-modified OligodT (/5Biosg/AAGCAGTGGTATCAACGCAGACATTTAGG (30T*V*(N)) and biotin-modified Random nonamer (/5Biosg/AAGCAGTGGTATCAACGCAGACATTTAGG NNNNNNNNN) primers, utilising an adapted Smartseq assay that targets all RNA transcripts.

Briefly, the first strand of the cDNA was synthesised with the addition of a few untemplated C nucleotides. This poly(C) overhang is added exclusively to full-length transcripts. The Transfer Switching Oligo (TSO) (AAGCAGTGGTATCAACGCAGAGTACATrGrG + G) was hybridised to the poly(C) overhang and used to synthesise the second strand. The full-length cDNAs were further amplified using KAPA HIFI HotStart Ready Mix (KAPA), purified, and quantified with the Promega Quantus Fluorometer (Promega Inc.).

ONT-compatible ready libraries were prepared using double-stranded cDNA with the Native Barcoding Kit 96 V14 SQK-NBD114.96, following the kit protocol. The prepared libraries were sequenced on the ONT PromethION 24 for up to 72 h using R10.4.1 flow cells and super accurate (SUP) basecalling with Dorado 7.6.8 to generate FASTQ files.

### Data analysis, PD-specific genes, comparison to PPMI data

Raw FASTQ files were transferred to the Pawsey Supercomputer Centre, Perth, Western Australia, where the data analysis was performed. We used the EPI2ME workflow for whole-transcriptome analysis with standard parameters and GRCh38p14 as the reference. The following script was run on the High Performance Computer:

*nextflow run epi2me-labs/wf-transcriptomes--de_analysis--direct_rna--fastq 'wfParkinson/files’ --transcriptome_source 'precomputed’ --ref_annotation '/GRCh38p14/gencode.v48.primary_assembly.annotation.gtf’ --ref_transcriptome 'wfParkinson/GRCh38p14/gencode.v48.transcripts.fa’ --ref_genome 'wfParkinson/GRCh38p14/GRCh38.primary_assembly.genome.fa’ --sample_sheet 'wfParkinson/samplefiles.csv’ --igv--out_dir PD145directrnaPreCompHighMem1910 --threads 128 -profile singularity*.

Briefly, the entire transcriptome workflow starts with *salmon* quantification, and the output is then used in further analytical tools [30]. This standard process performs differential gene expression (DEG) analysis using *edger* [31]. It also carries out differential transcript expression analysis (DEX) with *DEXseq* and differential transcript usage (DTU) through the Dirichlet-multinomial model, as implemented in *DRIMSeq*, to estimate the precision parameter [32–35]. To identify genes showing overall signs of DTU and pinpoint the specific transcripts differentially used by these genes, a two-stage statistical procedure in *stageR* was employed. Additional data manipulation, analysis, and plotting were performed in RStudio using the *DESeq2*, *tidyr*, *dplyr,* and *ggplot2* packages. Functional enrichment analysis of the differentially expressed genes and transcripts was conducted using the packages *DOSE*, *ReactomePA*, and *clusterProfiler*. Moreover, using the NCBI Gene database and search phrases “Parkinson disease OR Parkinson’s disease” AND “*Homo Sapiens*”, a list of 849 genes associated with Parkinson’s disease was compiled, and their enrichment in our transcriptomic profile was assessed. Finally, our results were compared with transcriptomic analyses of the Parkinson’s Progression Markers Initiative (PPMI) cohort to independently validate our findings.

## Results

### The study cohort

The main features of the study participants are summarised in Table 1. The cohort is evenly balanced for sex and age, with 41% men in the PD group and 43% in the HC group. The average age of the PD group is 69.8 years, compared to 70.1 years in the HC group. The mean age of male PD participants is 69.8 years, while that of male HC participants is 70.1 years. For female participants, the mean age of PD patients is 69.5 years, and for HC patients, it is 68.4 years. Similarly, the average duration since PD diagnosis is 7.9 years, with 8.4 years for males and 7.7 years for females. The mean duration from symptom onset was 9.9 years, and the mean total UPRDS score was 47.6. Detailed information is provided in Table 1. Because the study cohort was well balanced for age and sex, we did not adjust for these factors in our statistical models. We confirmed the absence of bias related to age or sex by comparing the adjusted model with other adjusted models.

### Differential gene expression analysis

**FIGURE 1:**
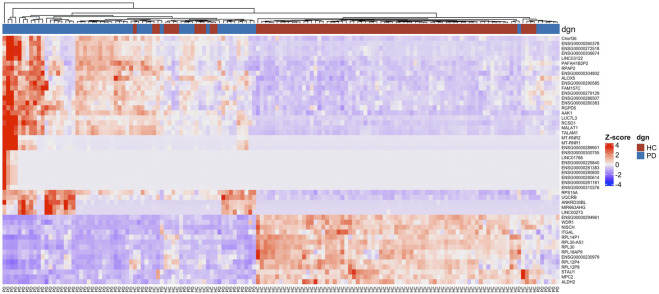
Heatmap of the top 50 differentially expressed genes with the lowest FDR values. HC - Healthy Control, PD - Parkinson’s Disease. The bar at the top of the heatmap indicates HC or PD.

**FIGURE 2:**
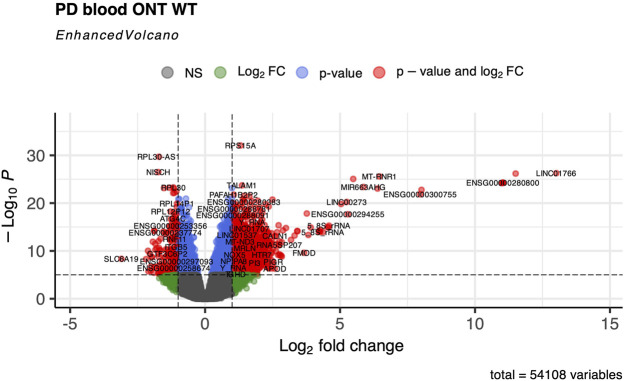
Volcano plot of the differentially expressed genes between PD patients and healthy controls.

Whole-transcriptome analysis identified 18,467 differentially expressed genes (DEGs) between PD and HC at a false discovery rate (FDR) < 0.05. An overview of the gene-expression differences is presented in Figures 1, 2, in Supplementary Table 1 and in Table 2 (for known Parkinson’s genes).

**TABLE 2:** Differentially expressed known Parkinson’s Disease genes between Parkinson’s patients and healthy controls.

Gene	logFC	logCPM	FDR	ENSEMBL gene Id	Description
MALAT1	1.439	12.9	6.22E-19	ENSG00000251562	Metastasis associated lung adenocarcinoma transcript 1
ALDH2	−0.95	5.9	2.41E-18	ENSG00000111275	Aldehyde dehydrogenase 2 family member
AAK1	0.852	6.92	2.42E-18	ENSG00000115977	AP2 associated kinase 1
GRN	2.025	5.55	7.11E-17	ENSG00000030582	Granulin precursor
LINC-PINT	0.986	6.02	4.97E-16	ENSG00000231721	Long intergenic non-protein coding RNA, p53 induced transcript
LRRK1	0.881	4.19	7.25E-15	ENSG00000154237	Leucine rich repeat kinase 1
MANF	−1.06	5	6.05E-14	ENSG00000145050	Mesencephalic astrocyte derived neurotrophic factor
PINK1-AS	−0.87	5.69	6.35E-14	ENSG00000117242	PINK1 antisense RNA
SRRM2	0.788	6.77	1.44E-13	ENSG00000167978	Serine/arginine repetitive matrix 2
PSEN1	0.548	7.14	2.18E-13	ENSG00000080815	Presenilin 1
RIC3	1.456	2.72	1.80E-12	ENSG00000166405	RIC3 acetylcholine receptor chaperone
MC1R	1.56	3.48	2.85E-12	ENSG00000258839	Melanocortin 1 receptor
CALB1	1.537	1.22	1.26E-11	ENSG00000104327	Calbindin 1
TMC3-AS1	1.71	1.15	1.60E-11	ENSG00000259343	TMC3 antisense RNA 1
VPS13C	−0.76	6.75	2.04E-11	ENSG00000129003	Vacuolar protein sorting 13 homolog C
HERPUD1	1.013	6.12	2.20E-11	ENSG00000051108	Homocysteine inducible ER protein with ubiquitin like domain 1
RNF11	−1.14	5.12	3.14E-11	ENSG00000123091	Ring finger protein 11
POLG	−0.96	7.34	5.32E-11	ENSG00000140521	DNA polymerase gamma, catalytic subunit
MT-ND3	1.298	5.38	9.32E-11	ENSG00000198840	Mitochondrially encoded NADH:ubiquinone oxidoreductase core subunit 3
BCL2L11	−0.82	4.76	1.03E-10	ENSG00000153094	BCL2 like 11
PINK1	−0.86	7.11	1.17E-10	ENSG00000158828	PTEN induced kinase 1

The most notable DEGs include ribosomal proteins, long non-coding RNAs (lncRNAs), their antisense transcripts, and novel transcripts (Supplementary Table 1). For example, RPL30-AS1 and RPL30, or MALAT1 and TALAM1, were differentially expressed in patients with PD compared with healthy controls. The top 10 up-regulated genes (LINC01766, ENSG00000225840, ENSG00000280614, ENSG00000280800, ENSG00000281181, ENSG00000281383, ENSG00000310376, ENSG00000300755, MT-RNR1, MT-RNR2) exhibited log fold changes ranging from 13 to 6, primarily involving lncRNA genes and novel transcripts. Interestingly, four of these novel transcripts were recognised as “similar to YY1-associated myogenesis RNA 1 YAM1,” suggesting their potential role in myogenesis [36]. ENSG00000300755 is an antisense lncRNA of the protein phosphatase with EF-hand domain 2 (PPEF2). PPEF2 opposes PINK1-mediated mitophagy and thus has a direct connection to PD pathology [37]. MT-RNR1 is a mitochondrial gene that encodes the 12S ribosomal RNA, a component of the mitochondrial ribosome, and mutations in it are known to cause sensorineural deafness and have also been linked to Parkinson’s disease [38]. MT-RNR2 is a mitochondrial gene that encodes the 16S large subunit ribosomal RNA and the humanin polypeptide. Humanin peptide has a very strong anti-apoptotic activity and is known as a neuroprotective factor [39].

**FIGURE 3:**
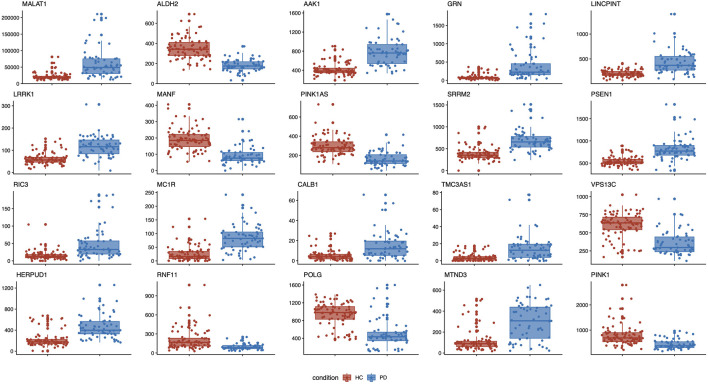
Boxplot showing known PD-related genes that are differentially expressed between PD patients and healthy controls. All genes in the boxplot have statistically significant differences.

**FIGURE 4:**
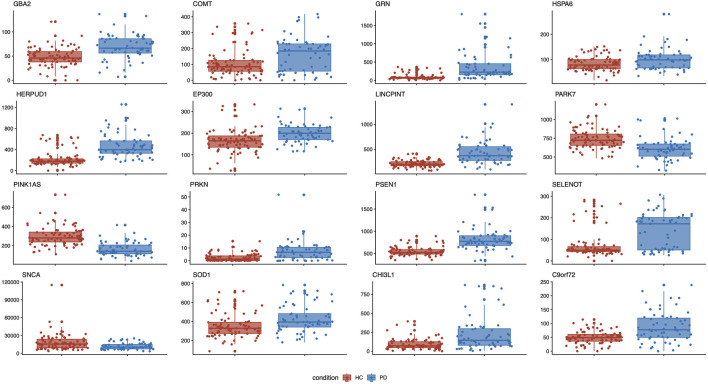
Boxplot showing the expression of genes that were differentially expressed identically in our sample and PPMI. All genes displayed in the boxplot have statistically significant differences.

The top down-regulated genes were SLC6A19, PACSIN3, THRAP3P3, ENSG00000287305, ENSG00000261065, TUBB2B, LINC02823, PMP22, SLC34A3, ENSG00000289158, BDNF and the logFC ranged from −3 to −1.9. The mutations in PMP22 cause Charcot-Marie-Tooth (CMT) disease. Many of these genes encode novel transcripts with unknown functions or no direct link to PD. However, BDNF is a gene with a well-defined function, and lower expression levels in PD patients have been reported previously [40]. The genes with a clear PD connection that appear at the top of the list of statistically significant differentially expressed genes are STAU1, AAK1, GRN and NPTN (Figure 3; Supplementary Table S2). STAU1 and NPTN were down-regulated, while GRN and AAK1 were up-regulated. The other well-known PD genes that showed statistically significant differences were SNCA, PARK7, and PINK1, all of which were down-regulated (Figure 4). A complete list of known PD genes, along with the 393 differentially expressed PD genes in our samples, is shown in the Supplementary Table 2.

### Comparison with the PPMI transcriptome analysis

We also compared our results with the analysis of the blood transcriptome from the PPMI cohort [16, 18]. We identified 3,283 common genes that are differentially expressed between PD and controls in both PPMI and our study (Figure 4; Supplementary Table 3). Of these DEGs, 2,994 are annotated and identified genes, while 289 are novel genes with unknown functions and names. Interestingly, GBA1 and LRRK2 were differentially expressed in PPMI but not in our ONT study.

We also observed that, out of the 3,283 common DEGs between PPMI and our sample, 145 genes were identified as PD-related (Supplementary Table 4). Remarkably, MMP9 was upregulated in both PPMI and ONT samples, and SNCA was downregulated in both samples.

### Differential transcript expression analysis

**FIGURE 5:**
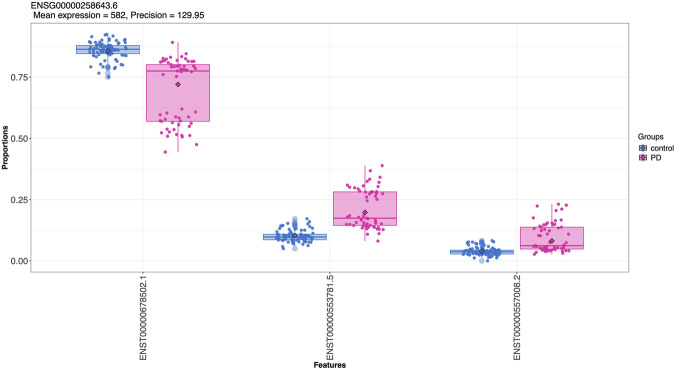
Differential transcript usage analysis. The figure shows the proportion of transcripts with differential expression for the gene BCL2L2-PABPN1. The most common transcript (ENST00000678502.1) is downregulated in PD patients, while two others (ENST00000553781.5, ENST00000557008.2) are upregulated.

For differential transcript analysis, *DEXseq*, *DRIMseq*, and *stager* were used. DEXseq analysis identified differential expression of 46,180 transcripts between PD and HC groups (Table 3; Supplementary Table 5). The analysis found three distinct BCL2L2-PABPN1 readthrough transcripts, all showing differential expression. The most common transcript was downregulated in PD, while two others were upregulated (Figure 5). Additionally, the gene ANKRD11 exhibited several transcripts with differential expression; two of these (ANKRD11-237, ANKRD11-224) are shown in Table 3. Furthermore, ribosomal protein S14 (RPS14) also displayed multiple differentially expressed transcripts (RPS14-206, RPS14-208). Among the most differentially expressed transcripts, sequestosome-1 (SQSTM1) and vacuolar protein sorting 13 homolog C (VPS13C) showed multiple transcripts with differential expression. Both genes are well recognised for their association with Parkinson’s disease.

**TABLE 3:** Differentially expressed transcripts between Parkinson’s patients and healthy controls.

Gene	LogFC	FDR	Description	Transcript name
BCL2L2-PABPN1	0.3905	2.57E-32	BCL2L2-PABPN1 readthrough	BCL2L2-PABPN1-201
CD82	−0.913	2.62E-32	CD82 molecule	CD82-204
ANKRD11	−1.194	9.94E-29	Ankyrin repeat domain containing 11	ANKRD11-237
RPS14	−1.412	9.94E-29	Ribosomal protein S14	RPS14-206
SPCS1	−1.078	9.94E-29	Signal peptidase complex subunit 1	SPCS1-204
RPL18A	−0.359	9.94E-29	Ribosomal protein L18a	RPL18A-202
TINF2	0.6424	2.28E-28	TERF1 interacting nuclear factor 2	TINF2-214
BCL2L2-PABPN1	−0.838	9.63E-28	BCL2L2-PABPN1 readthrough	BCL2L2-PABPN1-203
PABPC1	−0.69	9.70E-28	poly(A) binding protein cytoplasmic 1	PABPC1-240
RPS14	−0.36	2.75E-27	Ribosomal protein S14	RPS14-208
BRD7	−1.524	3.12E-27	Bromodomain containing 7	BRD7-207
RPL30	−0.838	3.12E-27	Ribosomal protein L30	RPL30-213
ANKRD11	−1.184	3.35E-27	Ankyrin repeat domain containing 11	ANKRD11-224
RPL29	−0.67	3.51E-27	Ribosomal protein L29	RPL29-203
SQSTM1	−0.743	4.94E-27	Sequestosome 1	SQSTM1-213
ACAP2	−0.485	4.94E-27	ArfGAP with coiled-coil, ankyrin repeat and PH domains 2	ACAP2-201
RPS23	0.6224	5.29E-27	Ribosomal protein S23	RPS23-204
PRR13	−0.586	6.41E-27	Proline rich 13	PRR13-207
RPL8	−0.555	1.86E-26	Ribosomal protein L8	RPL8-209
VPS13C	1.0497	2.18E-26	Vacuolar protein sorting 13 homolog C	VPS13C-210
TWF2	−0.744	3.26E-26	Twinfilin actin binding protein 2	TWF2-213

**FIGURE 6:**
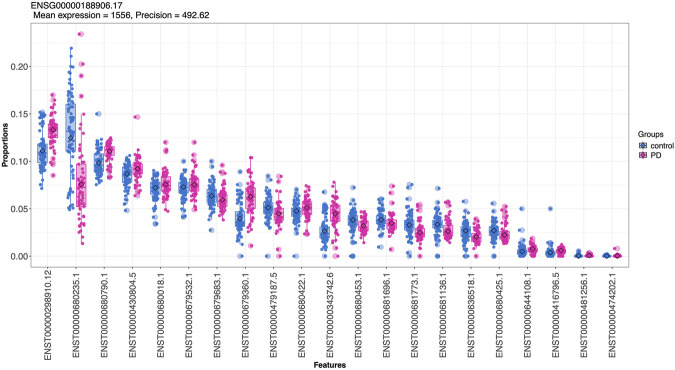
Differential transcript usage analysis. The figure displays the proportions of transcripts that are differentially expressed for LRRK2 (ENSG00000188906.17). Three transcripts are upregulated in PD patients, and one transcript is clearly downregulated.

**FIGURE 7:**
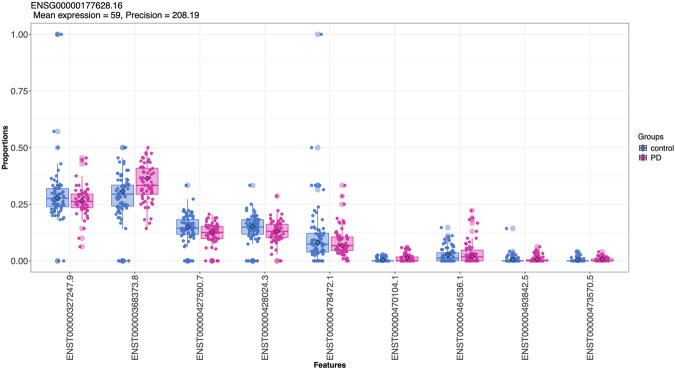
Differential transcript usage analysis. The figure illustrates the proportions of differentially expressed transcripts for GBA1 (ENSG00000177628.16). One transcript (ENST00000368373.8) is upregulated in PD patients.

Differential transcript usage (DTU) analysis is another method for identifying transcriptomic changes. This analysis compares the proportions of gene transcript isoforms across different conditions and highlights which genes are activated through transcript switching. Consequently, we identified 8,714 genes with statistically significant DTUs, and 35,360 transcripts exhibited statistically significant DTUs (Supplementary Table 6). DTU analysis also uncovered differential transcript usage for the GBA1 and LRRK2 genes (Figures 6, 7), although these genes were not differentially expressed at the gene level.

### Functional annotation of differential gene expression

**FIGURE 8:**
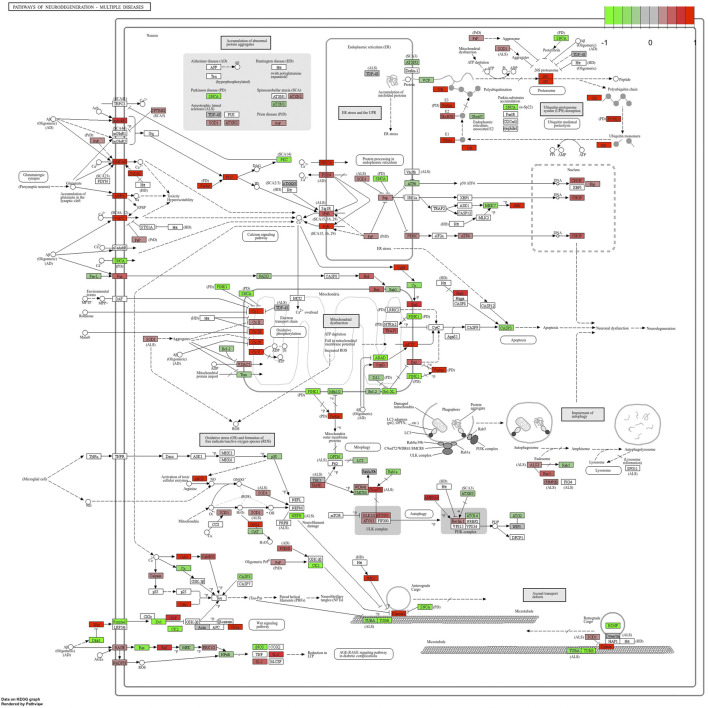
KEGG pathway analysis revealed several activated pathways in PD patients. This figure illustrates the activation of neurodegeneration-related pathways involved in multiple diseases. Genes in the mitochondria, lysosomes, and endoplasmic reticulum are affected in PD patients. Red colour indicates upregulation, and green colour indicates downregulation of the genes.

Functional annotation analysis of differentially expressed genes was conducted using the *clusterProfiler* and *ReactomePA* packages, which map log-fold-change values to predefined KEGG or Reactome pathways and perform formal statistical comparisons with FDR correction. We identified 72 KEGG pathways with FDR <0.05 (Supplementary Table 7). The ten most significantly enriched pathways are shown in Table 4. These top pathways are primarily associated with neurodegenerative diseases, such as Amyotrophic Lateral Sclerosis, Prion disease, and Parkinson’s disease (Figure 8). Additionally, several pathogenic molecular pathways (Protein processing in the ER, Oxidative phosphorylation) were enriched among the differentially expressed genes. Reactome enrichment analysis revealed 123 pathways that were significantly enriched (FDR <0.05). The most prominently enriched pathways (Supplementary Table 8) included mitochondrial energetics (Aerobic respiration and respiratory electron transport, Respiratory electron transport, Complex I biogenesis) and cellular damage (Neutrophil degranulation, Autophagy).

**TABLE 4:** The enrichment of KEGG pathways associated with differentially expressed genes.

pathwayID	Description	Fold enrichment	p-adjust
hsa05415	Diabetic cardiomyopathy	1.74	1.52E-12
hsa00190	Oxidative phosphorylation	1.91	1.52E-12
hsa05014	Amyotrophic lateral sclerosis	1.53	1.61E-12
hsa05020	Prion disease	1.61	5.48E-12
hsa05012	Parkinson disease	1.60	2.23E-11
hsa05208	Chemical carcinogenesis	1.62	2.17E-10
hsa05022	Multiple neurodegenerative diseases	1.41	2.35E-10
hsa05010	Alzheimer disease	1.46	2.56E-10
hsa05016	Huntington disease	1.49	4.10E-09
hsa04141	Protein processing in ER	1.59	3.47E-07

## Discussion

The present study employed Nanopore long-read sequencing to characterise whole-transcriptome changes in the blood of PD patients. This approach enables the accurate identification of full-length transcript isoforms without computational imputation and gives much clearer resolution of isoform diversity within whole transcriptomes, which often remains undetected or is only partially identified. It also enables the detection of transcript-level dynamics or differential transcript usage (DTU) associated with gene activation [30, 41]. Transcript isoform-level dynamics is especially important for neurodegenerative disorders, where the isoform diversity has already been suggested to drive the pathology [42, 43].

Previous studies have used short-read sequencing with Illumina or SOLiD technologies [7, 8, 16–19]. Although these technologies differ significantly, their outcomes show notable overlaps, with similar genes being upregulated or downregulated across studies. Typically, genes involved in the heat shock response, the ubiquitin-proteasome pathway, or the immune response are impacted [8]. Remarkably, SNCA has consistently been downregulated in PD patients in most transcriptomic analyses [18].

Different attempts have been made in the past to uncover the complexity of transcriptomic regulation in PD patients. One approach is to use short read data to analyse exonic and intronic transcription, which can provide some insight into isoform-level activation and RNA isoform switching [21, 22]. The intronic activation was evident when PD patients were compared to the controls, and also throughout the disease course, as thousands of introns were differentially expressed after 3 years of disease progression [21]. These previous studies clearly warrant deeper analysis of the PD transcriptome using nanopore technology, enabling more precise isoform detection.

Indeed, in the present study, we identified not only a large list of differentially expressed genes but also a huge number of differentially expressed transcripts between PD and controls. We identified 18,467 differentially expressed genes and 46,180 differentially expressed transcripts between the PD and HC groups (Table 3; Supplementary Table 5), including many novel transcripts and those that have never been linked to PD pathology. One of the most remarkable transcripts is the BCL2L2-PABPN1 readthrough, which combines two adjacent genes, BCL2L2 and PABPN1 and results in a fusion protein. While fusion proteins are more typical of cancer syndromes, the BCL2L2-PABPN1 has been found to be associated with obesity and metabolic rate [44]. The BCL2L2-PABPN1 gene was also differentially expressed in the gene-based analysis.

Another notable finding is the highly significant differential expression of the SQSTM1 transcripts. SQSTM1 encodes p62, a multifunctional adaptor protein that plays a central role in autophagy, proteostasis, cell signalling, inflammation, and bone metabolism. p62 acts as a scaffold protein that binds ubiquitinated cargo and delivers it to the autophagosome via its LC3-interacting region (LIR), making it a key receptor in selective autophagy pathways. SQSTM1 is involved in many neurodegenerative diseases, such as motor neurone disease (MND), Alzheimer’s disease, and Parkinson’s disease. Similarly, we observed differential expression of transcripts for the PABPC1 (poly(A) binding protein cytoplasmic 1) gene, which is involved in the pathogenesis of MND. Changes in PABPC1 localisation and expression have been linked to amyotrophic lateral sclerosis (ALS) through interactions with TDP-43 and FUS and stress granule dysfunction disorders. Its function links translational dysregulation to neuronal vulnerability [45, 46].

We also observed differential expression of VPS13C (Vacuolar Protein Sorting 13 Homolog C) at both gene and transcript levels. Interestingly, VPS13C was also identified in the previously mentioned study through intronic analysis [21]. VPS13C is essential for maintaining lipid balance, supporting lysosomal and mitochondrial functions, and managing cellular stress responses [47]. Mutations or disruptions in VPS13C are strongly linked to early-onset Parkinson’s disease, with additional connections to metabolic, inflammatory, and neurodegenerative disorders [48].

Based on the NCBI Gene database, we compiled a list of 849 genes associated with PD. This list defines PD-genes for our study, and although this definition is somewhat broad, it is inclusive and helps capture signals that might otherwise be missed in the analysis of our transcriptomic profile. We found that 393 genes with clear PD connections were differentially expressed.

One of such genes was AAK1 (Adaptor-Associated Kinase 1). AAK1 is linked to PD because it affects endocytosis and protein aggregation, both of which are crucial in the disease’s progression [49–51].

Furthermore, we compared this list of DEGs with published results for the PPMI DEGs and found that 145 genes were identically altered in the blood of PD patients. Some examples of the overlapping genes between the PPMI and our study include SNCA, DNAJB6, PINK1-AS, SELENOT, GRN, PARK7, and CHI3L1. More specifically, SNCA was downregulated in patients with PD, a finding reported by others.

SELENOT (Selenoprotein T) was upregulated in both PPMI and our study, making it an interesting gene for PD. SELENOT is a highly conserved selenoprotein containing selenocysteine, which allows for strong redox regulation and protection against oxidative stress [52]. It is primarily located in the endoplasmic reticulum (ER), where it plays vital roles in protein folding, ER homeostasis, calcium regulation, and cellular stress responses. Essential for cell survival and development, knockout models show embryonic lethality, highlighting its critical physiological function [53, 54]. SELENOT supports antioxidant defences, neuroendocrine regulation, and protection from ER stress, and has been linked to metabolic diseases, neurodegeneration, and inflammation. Its thioredoxin-like redox motif drives its molecular activity, positioning it as a key regulator of cellular balance. The strong ER-protective and antioxidant properties of SELENOT are especially relevant in conditions involving oxidative stress and protein misfolding, such as Parkinson’s, Alzheimer’s, and ALS/MND. Lower levels of SELENOT increase neuronal susceptibility to ER stress, while experimental activation reduces apoptosis and promotes neuronal survival. Additionally, its role in calcium homeostasis helps maintain synaptic integrity and provides neuroprotection [55].

GRN (granulin precursor) was also upregulated in PPMI and our study. GRN encodes progranulin, a secreted, multifunctional growth factor that supports cell survival, lysosomal activity, inflammation regulation, and tissue repair. Progranulin can be split into smaller granulin peptides, each with unique biological functions. It is highly expressed in the brain, immune system, and epithelial tissues, where it aids in maintaining neurons, activating microglia, healing wounds, and managing protein stability [56]. Haploinsufficiency of GRN is a major factor in frontotemporal dementia (FTD), leading to a gradual loss of progranulin and consequent lysosomal issues. Changes in GRN activity are also associated with neuroinflammation, Parkinson’s disease, Alzheimer’s disease, autoimmune disorders, and various cancers [56]. Due to its dual role in inflammation and growth factor signalling, GRN is a critical gene in neurodegeneration and immune regulation.

PARK7 was downregulated in both studies, and this is a well-known PD-related gene.

LRRK2 and GBA1 were not differentially expressed in gene-based analysis, but they became statistically significant when we analysed specific transcripts. Indeed, both genes showed significant transcript switching in PD patients compared to controls. This indicates the importance of analysing the transcriptome at the transcript level, in addition to traditional gene-level analysis. Interestingly, LRRK1 and GBA2 were also differentially expressed in the gene-based analysis. LRRK1 is a paralog of LRRK2, and while there have been no LRRK1 mutations linked to PD, the functional similarity between LRRK1 and LRRK2 suggests that LRRK1 might have some impact on the PD pathology. GBA2 (Glucosylceramidase 2) encodes a non-lysosomal β-glucosidase that helps break down glucosylceramide on the cytoplasmic side of the endoplasmic reticulum and Golgi apparatus. While GBA1 is the main gene associated with PD (due to lysosomal glucocerebrosidase deficiency), increasing evidence suggests that GBA2 also impacts sphingolipid levels and might influence PD risk and its underlying mechanisms.

We would also like to comment on our study design. PD is closely associated with ageing and shows known sex differences in immune and gene expression profiles, which can confound blood-based transcriptomic studies. To minimise the impact of these demographic factors, we designed a case–control study that matched participants by age- and sex-specific proportions. This approach aimed to prevent demographic confounding early on, reducing the need for extensive *post hoc* adjustments. This is particularly important for long-read Oxford Nanopore RNA sequencing, where age-related changes in transcript structures, isoform usage, and immune gene expression can be complex and not fully captured by statistical covariates. Using a matched cohort allows downstream analysis to focus on disease-specific transcriptional differences under similar demographic conditions. We acknowledge that this matching may reduce the effective sample size and limit generalisability, but it was chosen to improve internal validity and clarity in this exploratory transcriptomic study. When necessary, additional analyses, including age and sex as covariates, were performed to confirm the robustness of the results.

The main limitations of our study are the cross-sectional design and the absence of data on environmental factors. A cross-sectional approach was unavoidable due to the sampling process. Simultaneously, accurately collecting environmental data is quite challenging. For future studies, one environmental factor to consider is the season when blood samples are collected. The response to medication and quality of life in Parkinson’s patients are also affected by the seasons [57]. Although the exact mechanism remains unclear, seasonal fluctuations have been shown to influence behaviour and the endocrine system even in experimental settings [58, 59]. Additionally, the availability of the dopamine transporter depends on sunlight exposure, as shown in the PPMI cohort [60]. Likewise, several studies have demonstrated the link between seasons and sleep disturbances [61, 62].

On the other hand, our sample size is large enough to provide sufficient power for a thorough analysis. The high statistical power is reflected in the extensive list of differentially expressed genes and transcripts identified. However, for our future studies, more stratified analyses would be helpful for separating patients by genetic mutations or disease progression.

## Data Availability

The original contributions presented in the study are included in the article/Supplementary Material, further inquiries can be directed to the corresponding author.
